# Meta-analysis of the association between second-hand smoke exposure and ischaemic heart diseases, COPD and stroke

**DOI:** 10.1186/s12889-015-2489-4

**Published:** 2015-12-01

**Authors:** Florian Fischer, Alexander Kraemer

**Affiliations:** Department of Public Health Medicine, School of Public Health, University of Bielefeld, P.O. Box 100 131, 33501 Bielefeld, Germany

**Keywords:** Meta-analysis, Second-hand smoke, SHS, Ischaemic heart disease, COPD, Stroke

## Abstract

**Background:**

Second-hand smoke (SHS) is the most important contaminant of indoor air in first world countries. The risks associated with SHS exposure are highly relevant, because many people are regularly, and usually involuntarily, exposed to SHS. This study aims to quantify the effects of SHS exposure. Therefore, its impact on ischaemic heart diseases (IHD), chronic obstructive pulmonary diseases (COPD) and stroke will be considered.

**Methods:**

A systematic literature review was conducted to identify articles dealing with the association between SHS and the three outcomes IHD, COPD and stroke. Overall, 24 articles were included in a meta-analysis using a random effects model. Effect sizes stratified for sex and for both sexes combined were calculated.

**Results:**

The synthesis of primary studies revealed significant effect sizes for the association between SHS exposure and all three outcomes. The highest RR for both sexes combined was found for COPD (RR = 1.66, 95 % CI: 1.38–2.00). The RR for both sexes combined was 1.35 (95 % CI: 1.22–1.50) for stroke and 1.27 (95 % CI: 1.10–1.48) for IHD. The risks were higher in women than in men for all three outcomes.

**Conclusions:**

This is the first study to calculate effect sizes for the association between SHS exposure and the disease outcomes IHD, COPD, and stroke at once. Overall, the effect sizes are comparable with previous findings in meta-analyses and therefore assumed to be reliable. The results indicate the high relevance of public health campaigns and legislation to protect non-smokers from the adverse health effects attributable to SHS exposure.

**Electronic supplementary material:**

The online version of this article (doi:10.1186/s12889-015-2489-4) contains supplementary material, which is available to authorized users.

## Background

Second-hand smoke (SHS) still remains the most important contaminant of indoor air in first world countries [1]. Despite significant reductions within the past decades, a considerable part of the global population is regularly, and usually involuntarily, exposed to SHS. Therefore, it is a highly important risk factor for the total population. SHS exposure may lead to several chronic conditions, which are highly relevant in terms of morbidity and mortality for a population’s health [2]. There is a broad scientific consensus that SHS exposure is linked to carcinogenesis, in particular lung cancer. Furthermore, SHS has been linked to most diseases which are caused by active smoking [3–7]. This association is comprehensible due to the more than 50 carcinogens that have been identified in SHS [8].

Several mechanisms may lead to an increased likelihood of adverse effects in the cardiovascular and respiratory system. These mechanisms may cause a reduction in vascular flow and therefore the development of atherosclerosis [8, 9]. The mechanisms by which SHS exposure increases the risk of heart disease are multiple and interact with each other [10]. In comparison with lung cancer, there is one important difference in the association between SHS exposure and ischaemic heart diseases (IHD): for lung cancer, adverse health effects result from long-term exposure, whereas for other diseases, such as IHD, these effects are not merely long-term and chronic but also acute [11–15]. The effects of even brief passive smoking are often nearly as great as (chronic) active smoking [10, 16, 17].

### Evidence of adverse health effects attributable to SHS exposure

Research focused on the associations between SHS exposure and lung cancer first [18]. But subsequently other outcomes, such as IHD [19–21], respiratory diseases [22, 23] and stroke [24–26] were also included in the research. Beginning in 1984, observational studies started to point out the association between SHS exposure and IHD. This seems to be the most important outcome attributable to SHS exposure, because the effects on cardiovascular diseases are obvious even at low doses of SHS exposure [19, 27] and because IHDs are much more frequent than lung disease. Because IHD is so prevalent, even a small increase in risk associated with SHS exposure will have a substantial public health impact [28]. Extensive epidemiological research spanning a period of 25 years has indicated that SHS exposure increases the risk of IHD by 25-30 % [2, 10, 17, 19–21, 29], and this was also concluded by the Institute of Medicine [30]. The effects still remain if other factors such as dietary intake, socio-economic status, and health-care use are included in the analysis [31].

Furthermore, a dose–response relationship between the level of SHS exposure and the occurrence of IHD was observed [32]. The reported RR of 1.3 (indicating a 30 % excess risk) for the association between SHS exposure and IHD that has been described in several meta-analyses [12, 19, 20, 33, 34], is quite large compared to active smoking. The excess risk for regular SHS exposure is about one third of that smoking 20 cigarettes per day, although the total exposure to tobacco smoke is only 1 % of that from 20 cigarettes per day [4, 32]. Assuming a linear dose–response relationship would lead to an expected excess risk associated with SHS exposure of only 0.8 % (1 % of the 80 % excess risk from smoking 20 cigarettes per day) [35].

Active smoking is the most important risk factor for chronic obstructive pulmonary diseases (COPD). Almost 85-90 % of COPD related mortality is attributable to active cigarette smoking. However, it is also suggested that 10-15 % of COPD cases are attributable to other risk factors such as SHS exposure, occupational exposures, and genetic factors [22, 36]. Since environmental tobacco smoke contains potent airway irritants, SHS exposure could lead to chronic airway irritation, inflammation, and obstruction [37, 38]. Nevertheless, up to now the causal association between SHS exposure and COPD has received limited attention in epidemiological studies. The first studies focusing on the association between SHS exposure and COPD faced several limitations. First of all, most studies are based on self-reports and secondly, different methods for defining COPD were used. Therefore, the reported effects of passive smoking on lung function are small and partially inconsistent [22, 39–41].

Comparable to COPD, the relationship between SHS exposure and stroke was not verified for a long time [8, 42, 43]. In 2014, stroke was included as a condition that is causally linked to SHS exposure in the Surgeon General’s Report [44]. After several studies provided overall inconsistent results regarding the association between SHS exposure and stroke [25, 26, 43, 45–48], a meta-analysis of 20 studies indicated a strong dose-dependent association between SHS exposure and stroke [49].

### Study objective and research question

Tobacco use is one of the most important modifiable risk factors for several adverse health effects. Nevertheless, the effects of SHS exposure on health have not yet been fully recognized in public health policies [31, 50]. Although several studies have accounted for the (causal) associations between SHS exposure and disease conditions, some results are still inconsistent. In order to implement demand-actuated and successful strategies to protect the public from adverse health effects attributable to SHS exposure, it is necessary to provide evidence-based information about the magnitude and reliability of associations between SHS exposure and health outcomes. Therefore, this study aims to quantify the effect sizes of SHS exposure for three major outcomes: IHD, COPD, and stroke. Based on the results of a systematic review, a meta-analysis was performed to summarize the results of single studies in one effect size for each of the three outcomes. The main goals of the meta-analysis were: 1) to test whether the study results are homogeneous and, if so, 2) to obtain a combined estimator of the effect magnitude for the association between SHS exposure and the outcomes IHD, COPD and stroke. Although some meta-analyses have dealt with the association between SHS exposure and IHD as well as stroke, this is the first meta-analysis on the association between SHS exposure and COPD. Furthermore, it is the first study that allows a comparison of the effects for the selected outcomes, because the same methodology was used for the systematic literature review and meta-analysis.

## Methods

### Systematic literature review

As a first step, a systematic literature review was performed in PubMed according to the procedure and requirements described in the Preferred Reporting Items for Systematic Reviews and Meta-Analyses (PRISMA) statement [51]. The aim of the systematic review was to identify articles dealing with the association between SHS and the three outcomes (IHD, COPD, and stroke). All relevant literature in English or German language was included without any restrictions regarding the year of publication. The search was restricted to studies on the effects of SHS exposure in humans. The search in PubMed was completed in July 2015. Therefore, the systematic literature review contained articles published between 1984 and 2014. The following search algorithm was performed:

(second hand smok* [Title/Abstract] OR second-hand smok* [Title/Abstract] OR passive smok* [Title/Abstract] OR “tobacco smoke pollution” [Title/Abstract] OR environmental tobacco smok* [Title/Abstract]) AND (heart disease* [Title/Abstract] OR COPD [Title/Abstract] OR chronic obstructive pulmonary disease* [Title/Abstract] OR obstructive pulmonary disease* [Title/Abstract] OR chronic obstructive airways disease* [Title/Abstract] OR COAD [Title/Abstract] OR chronic obstructive lung disease* [Title/Abstract] OR COLD [Title/Abstract] OR stroke*[Title/Abstract] OR apople*[Title/Abstract])

Using the search algorithm under the above-mentioned filters led to the identification of 403 records. Among them, 221 were attributable to a combination of the search terms regarding exposure and the outcome IHD, 178 further articles were attributable to the search terms on COPD and 47 on stroke.^1^ After the screening of title and abstract, 307 of these articles were excluded, because they did not fit the study’s objective. Therefore, 96 full-texts were assessed for eligibility. According to this assessment, 71 articles were excluded for the following reasons^2^:

- study design
  - survey/cross-sectional study (9)
  - (systematic) review (28)
  - meta-analysis (5)
- no effect sizes provided (24)
- other outcomes observed (5)
- other exposures considered (4)
- letter to the editor (2)
- conflict of interest (1)

**Fig. 1:**
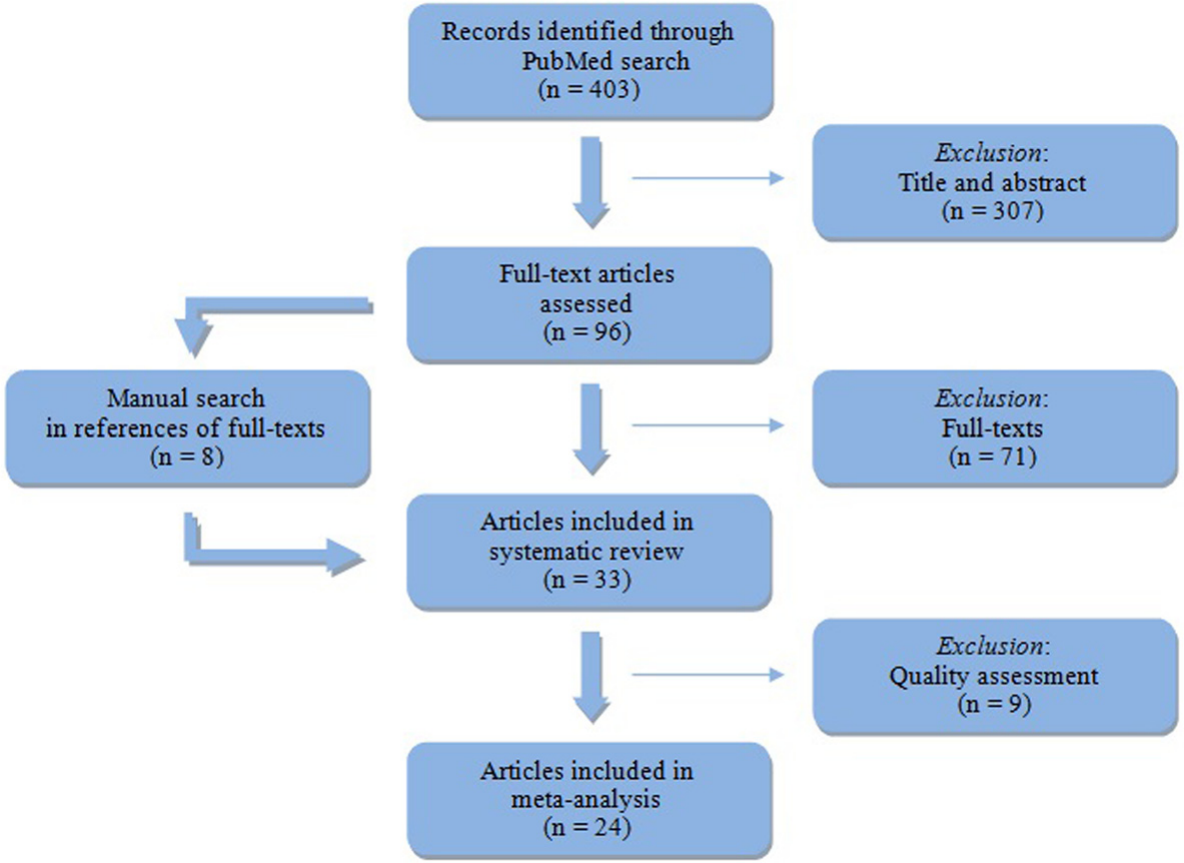
Flow chart for study selection

A manual search was conducted through the reference lists of all full-texts, which led to the inclusion of eight further articles. Finally, 33 articles were included in the qualitative analysis of the systematic review. Before including the studies in the quantitative synthesis in the form of a meta-analysis, a quality assessment was conducted. This quality assessment, which is described in more detail in the following section, led to the exclusion of further 9 studies. The process of the systematic review is presented in a flow chart (Fig. 1).

### Quality assessment

A checklist for the quality assessment was compiled on the basis of already existing and well-established instruments, such as the PRISMA guidelines [51] and instruments developed for observational studies [52–54]. The quality score developed for this study consists of three categories, with four items each. The first category was introduced to identify a selection bias. Therefore, the selection of cases and response rate are focused here. Since both case–control and cohort studies were included in the systematic review, two quality scales were developed which differed slightly in the aspects regarding recruitment of the study population. The second category deals with the assessment of misclassification bias. It is asked 1) whether the exposure evaluation was made in relation to the time of diagnosis, 2) whether the exposure was validated by a biomarker, 3) whether specific disease criteria were provided, and 4) whether the disease was validated by histology or another gold standard. The third category focuses on aspects of data analysis. One item was integrated to detect whether or not an adjustment of variables was performed. Additionally, studies with power calculations and sufficient sample size scored higher. A sample size was defined a priori as sufficient if at least 100 subjects were included in the analysis and a minimum of 20 cases occurred, in order to exclude studies with low precision. The last criterion was about the provision of exact *p*-values and confidence intervals (CI).

Each item of the quality score answered with “yes” received one point, and all items with the labels “uncertain/not reported” or “no” received no points. All points were summed which allows a maximum score of 12 points. A priori, it was decided that all studies with an overall score of 7 points or lower (*n* = 9) would be excluded from the meta-analysis.

### Calculation of relative risks

To allow for comparability between the results of the single studies, those results in which regular SHS exposure was investigated were focused upon. The definition of regular exposure varied between studies. Most commonly, spousal smoking or being exposed to about 20 cigarettes or more per day was interpreted as regular SHS exposure. In case studies divided between SHS exposure at home or at work, only the results for exposure at home were chosen. Nevertheless, several studies only provided information for SHS exposure at home and work combined.

The RR from the cohort studies were directly transferred to the summary of studies presented in Table 1. For case–control studies RR had to be derived from the provided odds ratios (OR). This was done for reasons of comparability of the results and because a single measurement unit was needed for the meta-analysis. For the calculation of RR based on OR an approach introduced by Barendregt [55] was selected. This approach describes the OR as a function of the RR, the average risk of disease in the population (s), and the prevalence of the risk factor (p). The equation uses the assumptions of the common definitions of RR and OR, and the observation that the average risk of a disease in any population is a linear combination of the risk in the exposed and non-exposed sub-populations:

$$ \mathrm{OR}=\frac{\mathrm{RR}\cdot \left(1-\frac{\mathrm{s}}{\mathrm{p}\cdot \mathrm{R}\mathrm{R}+1-\mathrm{p}}\right)}{1-\frac{\mathrm{RR}\cdot \mathrm{s}}{\mathrm{p}\cdot \mathrm{R}\mathrm{R}+1-\mathrm{p}}} $$

**Table 1:** Systematic literature review–Overview of all studies

*Nr*.	*Authors*	*Type*	*Location*	*Population*/*Participants*	*Exposure measurement*	*Exposure*	*Relative Risk*	*Controlled variables*	*Score*
							(*95* % *CI*)		
[26]	Bonita et al. (1999)	case–control	New Zealand	521 patients (279 men, 242 women) 1,851 controls (934 men, 917 women)	self-report	home and workplace	*stroke*	Yes	9
							1.65 (1.28–2.16)		
							men: 1.87 (1.27–2.77)		
							women: 1.53 (1.06–2.2)		
[72]	Chan-Yeung et al. (2007)	case–control	Hong Kong	289 patients (243 men, 46 women), 289 controls (243 men, 46 women)	self-report	home and workplace	*COPD*	Yes	9
							1.64 (0.97–2.03)		
[69]	Ciruzzi et al. (1998)	case–control	South America	336 patients (156 men, 180 women) 446 controls (228 men, 218 women) *never*-*smokers*	self-report	home	*IHD*	Yes	9
							2.04 (0.99–12.52)		
[16]	Ding et al. (2009)	case–control	Hong Kong	314 female patients, 319 female controls, *never*-*smokers*	self-report	home: ≥ 4 h/day	*IHD*	Yes	9
							women: 1.31 (1.03–6.01)		
[87]	Dobson et al. (1991)	case–control	Australia	759 patients, (519 men, 240 women) 1,308 controls (625 men, 683 women) *non*-*smokers*	self-report (medical records and relatives for deaths)	home and workplace	*IHD*	Yes	9
							men: 0.98 (0.63–1.33)		
							women: 1.92 (1.33–2.69)		
[88]	Gallo et al. (2010)	cohort	Europe	135,233 (19,922 men, 115,311 women) *never*-*smokers*	self-report	home	*stroke*	Yes	6
							men: 1.10 (0.36–3.37)		
							women: 0.93 (0.49–1.74)		
[48]	Glymour et al. (2008)	cohort	USA	16,225 *never*-*smokers*	self-report	home: spousal smoking (current exposure)	*stroke*	Yes	9
							1.42 (1.02–1.92)		
							men: 1.63 (0.83–2.70)		
							women: 1.46 (1.00–2.18)		
[66]	He et al. (1994)	case–control	China	59 female patients, 126 female controls, *never*-*smokers*	self-report	home	*IHD*	Yes	10
							women: 1.16 (0.67–1.95)		
[89]	He et al. (2012)	cohort	China	910 (439 men, 471 women) *never*-*smokers*	self-report	home and workplace	*IHD*	Yes	7
							2.15 (1.00–4.61)		
							men: 2.24 (0.76–6.59)		
							women: 2.10 (0.69–6.33)		
							*COPD*		
							2.30 (1.06–5.00)		
							men: 2.15 (0.86–5.39)		
							women: 3.31 (0.69–15.82)		
							*stroke*		
							2.22 (1.21–4.10)		
							men: 2.25 (1.09–4.66)		
							women: 2.02 (0.62–6.53)		
[28]	Helsing et al. (1988)	cohort	USA	19,035 (4,162 men, 14,873 women) *never*-*smokers*	self-report	home: spousal smoking	IHD	Yes	7
							men: 1.38 (1.1–1.8)		
							women: 1.20 (1.0–1.4)		
[90]	Hill et al. (2007)	cohort	New Zealand	381,462 (152,613 men, 228,849 women) *never*-*smokers*	self-report	home	HD	Yes	7
							men: 1.18 (0.96–1.44)		
							women: 1.27 (0.98–1.66)		
							*stroke*		
							men: 1.82 (1.20–2.77)		
							women: 1.17 (0.76–1.82)		
[91]	Hole et al. (1989)	cohort	Scotland	7,997 (3,960 men, 4,037 women)	self-report	home	*IHD*	Yes	8
							2.01 (1.21–3.35)		
[43]	Iribarren et al. (2004)	cohort	USA	27,698, (10,482 men, 17,216 women)	self-report	home: ≥ 20 h/week	*stroke*	Yes	8
							1.42 (1.08–1.88)		
							men: 1.29 (0.75–2.20)		
							women: 1.50 (1.07–2.09)		
[92]	Jefferis et al. (2010)	cohort	Great Britain	2,783 *never*-*smokers*	self-report and cotinine-assessment	home	*IHD*	Yes	6
							1.00 (0.86–1.16)		
							*stroke*		
							0.94 (0.80–1.11)		
[64]	Johannessen et al. (2012)	case–control	Norway	433 patients (258 men, 175 women) 325 controls, (176 men, 149 women)	self-report	home	*COPD*	No	7
							men: 0.98 (0.81–1.17)		
							women: 1.14 (0.93–1.37)		
[63]	Kalandidi et al. (1990)	case–control	Greece	103 female patients 179 female controls, *never*-*smokers*	self-report	home: spousal smoking (1–20 cigarettes/day)	*COPD*	No	7
							women: 1.79 (1.17–2.57)		
[31]	Kawachi et al. (1997)	cohort	USA	32,056 female nurses, *never*-*smokers*	self-report	home and workplace: regular exposure	*IHD*	Yes	8
							women: 1.91 (1.11–3.28)		
[70]	McElduff et al. (1998)	case–control	New Zealand/Australia	953 patients (686 men, 267 women), 3,189 controls, (1,559 men, 1,630 women) ≥ *10 years non*-*smokers*	self-report	home and workplace	*IHD*	Yes	9
							men: 1.01 (0.86–1.18)		
							women: 1.78 (1.33–2.36)		
[24]	McGhee et al. (2005)	case–control	Hong Kong	4,838 cases (2,680 men, 2,158 women) 763 controls (418 men, 345 women), *never*-*smokers*	self-report	home	*IHD*	Yes	9
							1.18 (1.02–1.36)		
							men: 1.15 (0.93–1.38)		
							women: 1.22 (0.97–1.53)		
							*COPD*		
							1.81 (1.24–2.65)		
							men: 1.50 (0.96–2.28)		
							women: 2.59 (1.30–5.27)		
							*stroke*		
							1.24 (1.08–1.42)		
							men: 1.16 (0.92–1.44)		
							women: 1.27 (1.06–1.53)		
[93]	Muscat and Wynder (1995)	case–control	USA	114 cases (68 men, 46 women) 158 controls (108 men, 50 women) *never*–*smokers*	self-report	home and workplace	*IHD*	Yes	8
							men: 1.06 (0.55–1.83)		
							women: 1.33 (0.71–2.87)		
[67]	Panagiotakos et al. (2002)	case–control	Greece	848 cases (700 men, 148 women) 1,078 controls (862 men, 216 women) *non*-*smokers*	self-report	home and workplace: regular exposure	*IHD*	Yes	10
							men: 1.43 (1.38–1.47)		
							women: 1.46 (1.41 – 1.51)		
[50]	Pitsavos et al. (2002)	case–control	Greece	848 cases (700 men, 148 women) 1,078 controls (862 men, 216 women) *non*-*smokers*	self-report	home: regular exposure	*IHD*	Yes	9
							1.17 (1.06–1.61)		
[45]	Qureshi et al. (2005)	cohort	USA	3,032 women *non*-*smokers*	self-report	home: spousal smoking	*stroke*	Yes	7
							0.8 (0.6–1.3)		
[68]	Rosenlund et al. (2001)	case–control	Sweden	334 cases (199 men, 135 women) 677 controls (401 men, 276 women) *never*-*smokers*	self-report	home: spousal smoking (current exposure)	*IHD*	Yes	8
							1.23 (0.93–1.57)		
							men: 0.99 (0.67–1.39)		
							women: 1.79 (1.17–2.54)		
[94]	Rostron (2013)	cohort	USA	7,586 *never*-*smokers*	cotinine-assessed	home: high exposure	*IHD*	Yes	8
							2.47 (1.04–5.86)		
[95]	Schwartz et al. (2009)	case–control	USA	562 female cases, 564 female controls	self-report	home	*COPD*	Yes	9
							women: 1.68 (1.12–2.61)		
[65]	Steenland et al. (1996)	cohort	USA	309,599 (101,227 men, 208,372 women) *never*-*smokers*	self-report	home: spousal smoking	*IHD*	Yes	7
							men: 1.22 (1.07–1.40)		
							women: 1.10 (0.96–1.27)		
[73]	Wen et al. (2006)	cohort	China	72,829 women *never*-*smokers*	self-report	home: spousal smoking (current exposure)	*IHD*	Yes	8
							women: 1.37 (1.06–1.78)		
							*stroke*		
							women: 1.52 (1.08–2.15)		
[96]	Whincup et al. (2004)	cohort	Great Britain	945 men *never*-*smokers*	cotinine-assessment	not specified	*IHD*	Yes	9
							men: 1.67 (0.91–3.07)		
[75]	Wu et al. (2010)	case–control	Taiwan	205 female cases 205 female controls	self-report (validation by cotinine-assessment for 71 subjects)	home and workplace	*COPD*	Yes	9
							women: 3.12 (1.56–6.50)		
[71]	Yin et al. (2007)	Cohort	China	15,379 (1,777 men, 13,602 women) *never*-*smokers*	self-report	home: ≥ 5 years of 40 h/week	*COPD*	Yes	8
							1.60 (1.23–2.10)		
[25]	You et al. (1999)	case–control	Australia	154 cases, 213 controls, *never*-*smokers*	self-report	home: spousal smoking (>20 cigarettes/day)	*stroke*	Yes	9
							1.44 (0.96–2.01)		
[74]	Zhang et al. (2005)	Cohort	China	60,377 women, *never*-*smokers*	self-report	home: spousal smoking (≥20 cigarettes/day)	*stroke*	Yes	8
							women: 1.62 (1.28–2.05)		

The reciprocal conversion from OR to RR requires a numerical optimization procedure. The detailed derivation of the equation and the Excel add-in for the calculation of RR is provided by Barendregt [55].

### Meta-analysis

The provided or calculated RRs from the primary studies with high methodological quality were used for the meta-analysis. The meta-analysis was conducted in MIX 2.0 Pro, which is a statistical add-in to perform meta-analysis with Microsoft Excel [56]. As a first step, the RRs and CIs from all the studies were converted into the logarithm function of the RR (log (rr)) and standard errors (se). This information, including the sample size, was used to calculate effect sizes for each of the three outcomes, stratified by sex. The precision was set to an alpha-level of 0.05 and a z-distribution as the standard distribution was chosen. For the analysis, a generic inverse-variance method random effects model was chosen, to provide estimates for the association between SHS exposure and the outcomes IHD, COPD and stroke. In this model, weight is given to each study according to the inverse variance of the effect, to minimize uncertainty about the summarized effect estimates, according to the widely used approach developed by DerSimonian and Laird [57].

### Statistical analysis

The random effects model was chosen, because the data were expected to be heterogeneous across studies. The advantage of a random effects model is that it incorporates variation in the underlying effect sizes between studies. It is assumed that each single study has its own (true) effect and that there is a random distribution of these effects around a central effect [58]. In contrast, using a fixed effect model under conditions of heterogeneity, the CI for the overall effects reflects the random variation within each study, but not the potential heterogeneity across studies, which would lead to artificially narrow CIs [59]. Furthermore, random effects models are more sensitive to publication bias, due to the larger relative weight given to smaller studies. This implies that a random effects model may still be worth considering as it cannot be assumed that true homogeneity exists across the studies [60].

In order to consider the sensitivity of results, potential publication and study bias were assessed visually using a heterogeneity funnel plot (see Additional file 1). Additionally, heterogeneity was quantified using two statistical measures: The Q- and I^2^-statistics reflect a certain dimension of the extent of heterogeneity between the studies. The Q-statistic is the sum of the weighted squared differences between each individual study’s estimate and the overall (inverse variance) summary estimates. This statistic follows a χ^2^-distribution with k–1 degrees of freedom, under the null-hypothesis of homogeneity. The Q-test is defined by Hedges and Olkin [61] as:

$$ \mathrm{Q}={\displaystyle \sum }{\mathrm{w}}_{\mathrm{i}}\cdot {\left({\mathrm{T}}_{\mathrm{i}}-\overline{\mathrm{T}}\right)}^2 $$

In this equation, *w*_*i*_ is the weighting factor for the *i*th study, *T*_*i*_ is the *i*th effect estimate in a collection of *k* studies and $\overline{T}$ is the estimate of the mean effect size, which consists of weighting every effect estimate T_i_ by its inverse variance. A *p*-value < 0.1 for the Q-statistic indicates heterogeneity [61].

Afterwards, the I^2^ is derived from the Q-statistic. The I^2^-index measures the extent of true heterogeneity by dividing the difference between the results of the Q test and its degrees of freedom by the Q-value itself, and multiplying by 100:

$$ {\mathrm{I}}^2=\frac{\mathrm{Q}-\left(\mathrm{k}-1\right)}{\mathrm{Q}}\cdot 100 $$

The I^2^-index quantifies the proportion of inconsistency among the study results. It is commonly expressed as a percentage and is therefore interpreted as the percentage of the total variability in a set of effect sizes due to between-study variation that is not attributable to random sampling from a fixed parameter [62]. Higgins and Thompson [62] proposed a tentative classification of I^2^-values to help in the interpretation of the heterogeneity’s magnitude: according to this classification, percentages of around 25 %, 50 % and 75 % would mean low, medium, and high heterogeneity, respectively.

## Results

### Studies of SHS exposure and selected outcomes

Overall, 33 studies were included in the systematic review. The first article was published in 1988, and the most recent in 2013. Several of the articles provided information on more than one outcome. Most articles described the effect of SHS exposure on IHD (*n* = 20). In 12 articles stroke was investigated as an outcome and eight articles focused on COPD (Table 1).

The spatial distribution of the study locations of all studies identified by the systematic review is quite equal: nine studies were performed in Asia (mainly in China and Hong Kong), Europe (mainly in Great Britain and northern European countries), and the USA. A further five studies were located in Australia and/or New Zealand and one in South America. Half of the articles described the results of a case–control study (*n* = 17) and the other half used a cohort design (*n* = 16). In almost all studies, information on SHS exposure was based on self-reporting (*n* = 30), while two studies performed a cotinine assessment for measuring SHS exposure and one study used a combination of self-reporting and cotinine assessment (Table 1). Usually, never-smokers or non-smokers were studied. However, some studies did not provide any information on the smoking status of subjects or included active smokers as well as non-smokers. In these cases, smoking status was controlled for in the analyses. All but two studies [63, 64] controlled for several factors.

The study samples varied between 309 599 never-smokers in a cohort study in the USA, dealing with the association between SHS exposure and IHD [65] and a case–control study with 56 female IHD patients and 136 female controls in China [66].

### Effect sizes for SHS exposure and selected outcomes

#### SHS and ischaemic heart disease

The RR for the single studies dealing with the association between SHS and IHD are presented in Table 2. From the 20 studies on IHD in the systematic review, five were excluded because of low methodological quality according to the quality assessment. Additionally, the Greek study from Panagiotakos et al. [67] was excluded in the meta-analysis, because the same data was used in the study by Pitsavos et al. [50], in which the analysis was stratified by place of exposure. This led to 14 studies on the effects of SHS exposure on IHD. In 6 of these studies, information summarized for both sexes were provided (*n* = 24 903). The RR for the association between SHS and IHD was either stratified by sex or only observed for one sex in six studies for men (*n* = 8208) and nine for women (*n* = 111 533).

**Table 2:** Effect sizes–SHS and ischaemic heart disease

*Nr*.	*Authors*	*Sex*	*RR* (*95* % *CI*)	*log* (*rr*)	*se*	*n*	*w* (%)
[69]	Ciruzzi et al. (1998)	both sexes	2.04 (0.99–12.52)	0.71	0.65	782	1.39
[16]	Ding et al. (2009)	women	1.31 (1.03–6.01)	0.27	0.45	633	2.27
[87]	Dobson et al. (1991)	men	0.98 (0.63–1.33)	−0.02	0.19	1,144	8.35
		women	1.92 (1.33–2.69)	0.65	0.18	923	12.19
[66]	He et al. (1994)	women	1.16 (0.67–1.95)	0.15	0.27	185	5.87
[91]	Hole et al. (1989)	both sexes	2.01 (1.21–3.35)	0.70	0.26	7,997	7.69
[31]	Kawachi et al. (1997)	women	1.91 (1.11–3.28)	0.65	0.28	32,056	5.72
[70]	McElduff et al. (1998)	men	1.01 (0.86–1.18)	0.01	0.08	2,245	46.59
		women	1.78 (1.33–2.36)	0.58	0.15	1,897	16.90
[24]	McGhee et al. (2005)	both sexes	1.18 (1.02–1.36)	0.17	0.07	5,601	38.73
		men	1.15 (0.93–1.38)	0.14	0.10	3,098	29.93
		women	1.22 (0.97–1.53)	0.20	0.12	2,503	23.48
[93]	Muscat and Wynder (1995)	men	1.06 (0.55–1.83)	0.06	0.31	176	3.23
		women	1.33 (0.71–2.87)	0.29	0.36	96	3.56
[50]	Pitsavos et al. (2002)	both sexes	1.17 (1.06–1.61)	0.16	0.11	1,926	27.88
[68]	Rosenlund et al. (2001)	both sexes	1.23 (0.93–1.57)	0.21	0.13	1,011	21.40
		men	0.99 (0.67–1.39)	−0.01	0.19	600	8.75
		women	1.79 (1.17–2.54)	0.59	0.20	411	10.37
[94]	Rostron (2013)	both sexes	2.47 (1.04–5.86)	0.90	0.44	7,586	2.91
[73]	Wen et al. (2006)	women	1.37 (1.06–1.78)	0.31	0.13	72,829	19.64
[96]	Whincup et al. (2004)	men	1.67 (0.91–3.07)	0.51	0.31	945	3.15
		*Sex*	*RR* (*95* % *CI*)	*Q*	*p*	*I* ^*2*^	
	Synthesis	both sexes	1.27 (1.10–1.48)	7.22	0.205	30.78	
		men	1.06 (0.96–1.19)	3.46	0.629	0.00	
		women	1.50 (1.31–1.72)	9.52	0.300	16.00	

The synthesis of all the studies included in the meta-analysis results in a RR of 1.27 (95 % CI: 1.10 – 1.48) for both sexes together. The RR was much higher for women (RR = 1.50, 95 % CI: 1.31 – 1.72) than for men (RR = 1.06, 95 % CI: 0.96 – 1.19). None of the studies showed significant results for men regarding the association between SHS exposure and IHD.

**Fig. 2:**
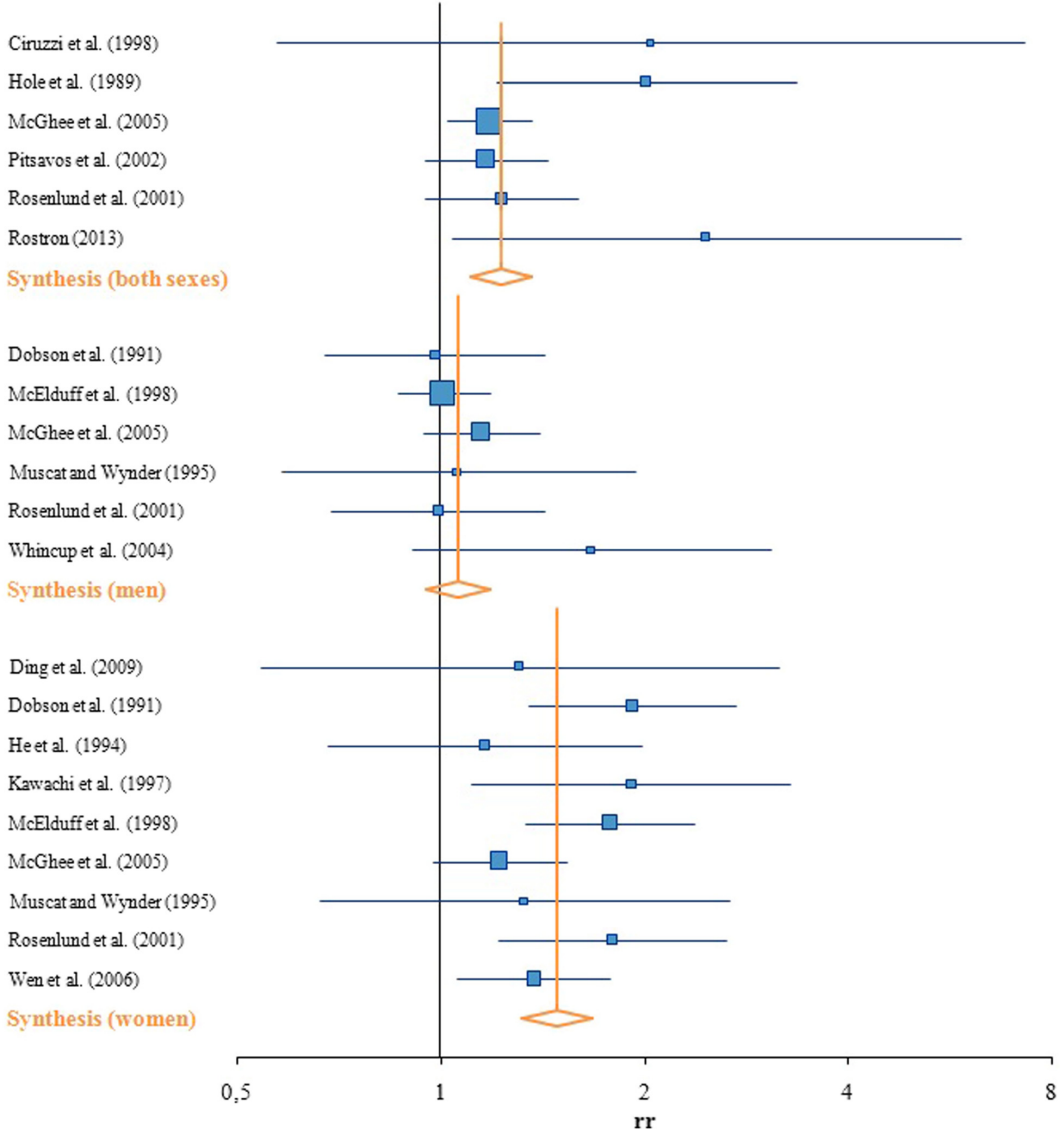
Forest plot–SHS and ischaemic heart disease

The studies from McGhee et al. [24], Pitsavos et al. [50] and Rosenlund et al. [68] had the highest impact on the synthesis, because these three studies were weighted with 88 % overall. The results of Ciruzzi et al. [69], with a very broad confidence interval (RR = 2.04, 95 % CI: 0.99–12.52), contributed only to a small extent to the overall RR due to the weighting factor of 1.39 %. For men, the study by McElduff et al. [70] contributed most to the synthesis result (46.59 %). For women, several studies contributed to more or less the same extent to the synthesis (Table 2, Fig. 2).

Cochran’s Q-test revealed no heterogeneity, because the *p*-value was larger than 0.1 for all three subgroup syntheses. This is confirmed by the I^2^-statistic, which quantifies the assumption between the three different subgroup syntheses. According to the results of these tests, no heterogeneity was observed for men (I^2^ = 0 %), and only a small but negligible heterogeneity for the studies focusing on women (I^2^ = 16.00 %). I^2^ was highest for studies including both sexes (I^2^ = 30.78 %), because the RR obviously differed for men and women (Table 2).

#### SHS and chronic obstructive pulmonary disease

Only five studies investigating the association between SHS exposure and COPD were included in the meta-analysis, after three further studies were excluded because of low quality. Overall, 28 965 participants were included in these studies, with more than half of them (*n* = 15 379) being investigated in one Chinese cohort study [71]. In three studies the RRs for the association between SHS and COPD were calculated for both sexes combined (*n* = 21,558). Only McGhee et al. [24] provided information stratified for men (*n* = 3,098) and women (*n* = 2,503) and two further studies investigated the association between SHS and COPD in a female-only study population (Table 3).

**Table 3:** Effect sizes–SHS and COPD

*Nr*.	*Authors*	*Sex*	*RR* (*95* % *CI*)	*log* (*rr*)	*se*	*n*	*w* (%)
[72]	Chan-Yeung et al. (2007)	both sexes	1.64 (0.97–2.03)	0.49	0.19	578	25.96
[24]	McGhee et al. (2005)	both sexes	1.81 (1.24–2.65)	0.59	0.19	5,601	24.55
		men	1.50 (0.96–2.28)	0.41	0.22	3,098	100.00^a^
		women	2.59 (1.30–5.27)	0.95	0.36	2,503	24.69
[95]	Schwartz et al. (2009)	women	1.68 (1.12–2.61)	0.52	0.22	1,126	51.40
[75]	Wu et al. (2010)	women	3.12 (1.56–6.50)	1.14	0.36	410	23.91
[71]	Yin et al. (2007)	both sexes	1.60 (1.23–2.10)	0.47	0.14	15,379	49.49
		*Sex*	*RR* (*95* % *CI*)	*Q*	*p*	*I* ^*2*^	
	Synthesis	both sexes	1.66 (1.38–2.00)	0.28	0.871	0.00	
		men	1.50 (0.96–2.28)^a^				
		women	2.17 (1.48–3.18)	2.60	0.273	22.95	

^a^synthesis for men only based on McGhee et al. (2005)

The large study by Yin et al. [71] accounted for almost half (49.49 %) of the weighting factor for both sexes. Two further studies, by Chan-Yeung et al. [72] and McGhee et al. [24], accounted for 25 % each for the weighting factor in the subgroup of both sexes. For the female subgroup, the weighting factors were distributed in a similar way for the three studies included, although based on different studies.

**Fig. 3:**
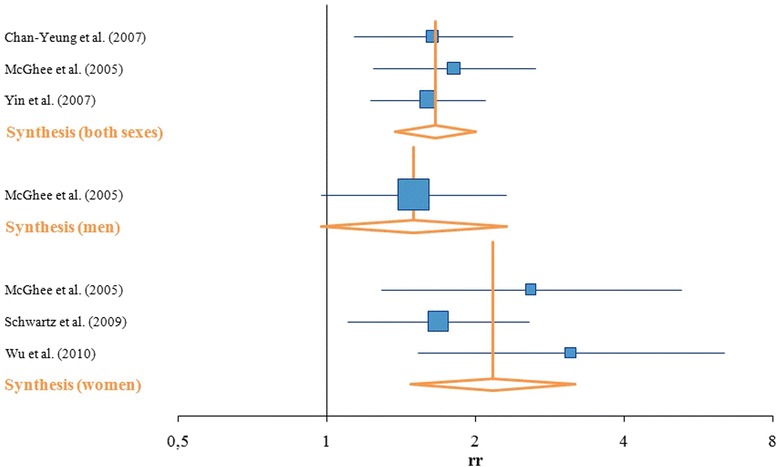
Forest plot–SHS and COPD

The synthesis for both sexes is based on three studies with consistent and significant results. A RR of 1.66 with a comparatively small confidence interval (95 % CI: 1.38–2.00) was calculated. Since the synthesis for men is based on only one study, the RR of 1.50 (95 % CI: 0.96–2.28) was inherited. For women, a higher RR was identified (RR = 2.17, 95 % CI: 1.48–3.18) than for men (Table 3, Fig. 3).

The heterogeneity between studies was assessed for the subgroups of both sexes and for women. The Q-statistic and its *p*-value suggested no heterogeneity between study results. The I^2^ for both sexes was 0 % and for women it was 22.95 %, which indicates no or only small heterogeneity (Table 3).

### SHS and stroke

The results for stroke are based on seven studies, after five studies were excluded due to the quality assessment. Five studies provided information combined for both sexes (*n* = 52,263). In four studies the analysis was stratified for sex. This leads overall to 22 905 male study participants. Two large additional studies focused only on women, which leads overall to 162 197 female study participants, which allows for investigating the association between SHS exposure and stroke. For the synthesis of all three subgroups, the study performed by McGhee et al. [24] is of particular importance due to its high weighting factor (Table 4).

**Table 4:** Effect sizes–SHS and stroke

*Nr*.	*Authors*	*Sex*	*RR* (*95* % *CI*)	*log* (*rr*)	*se*	*n*	*w* (%)
[26]	Bonita et al. (1999)	both sexes	1.65 (1.28–2.16)	0.50	0.13	2,372	15.23
		men	1.87 (1.27–2.77)	0.63	0.20	1,213	25.68
		women	1.53 (1.06–2.22)	0.43	0.19	1,159	9.27
[48]	Glymour et al. (2008)	both sexes	1.42 (1.02–1.92)	0.35	0.16	16,225	10.48
		men	1.63 (0.83–2.70)	0.49	0.30	8,112	14.39
		women	1.46 (1.00–2.18)	0.38	0.20	8,113	8.34
[43]	Iribarren et al. (2004)	both sexes	1.42 (1.08–1.88)	0.35	0.14	27,698	13.60
		men	1.29 (0.75–2.20)	0.25	0.27	10,482	16.56
		women	1.50 (1.07–2.09)	0.41	0.17	17,216	11.30
[24]	McGhee et al. (2005)	both sexes	1.24 (1.08–1.42)	0.22	0.07	5,601	52.98
		men	1.16 (0.92–1.44)	0.15	0.11	3,098	43.38
		women	1.27 (1.06–1.53)	0.24	0.09	2,503	37.59
[73]	Wen et al. (2006)	women	1.52 (1.08–2.15)	0.42	0.18	72,829	10.68
[25]	You et al. (1999)	both sexes	1.44 (0.96–2.01)	0.36	0.19	367	7.71
[74]	Zhang et al. (2005)	women	1.62 (1.28–2.05)	0.48	0.12	60,377	22.83
		*Sex*	*RR* (*95* % *CI*)	*Q*	*p*	*I* ^*2*^	
	Synthesis	both sexes	1.35 (1.22–1.50)	4.08	0.395	2.08	
		men	1.40 (1.09–1.81)	4.84	0.184	37.95	
		women	1.43 (1.28–.61)	3.02	0.697	0.00	

**Fig. 4:**
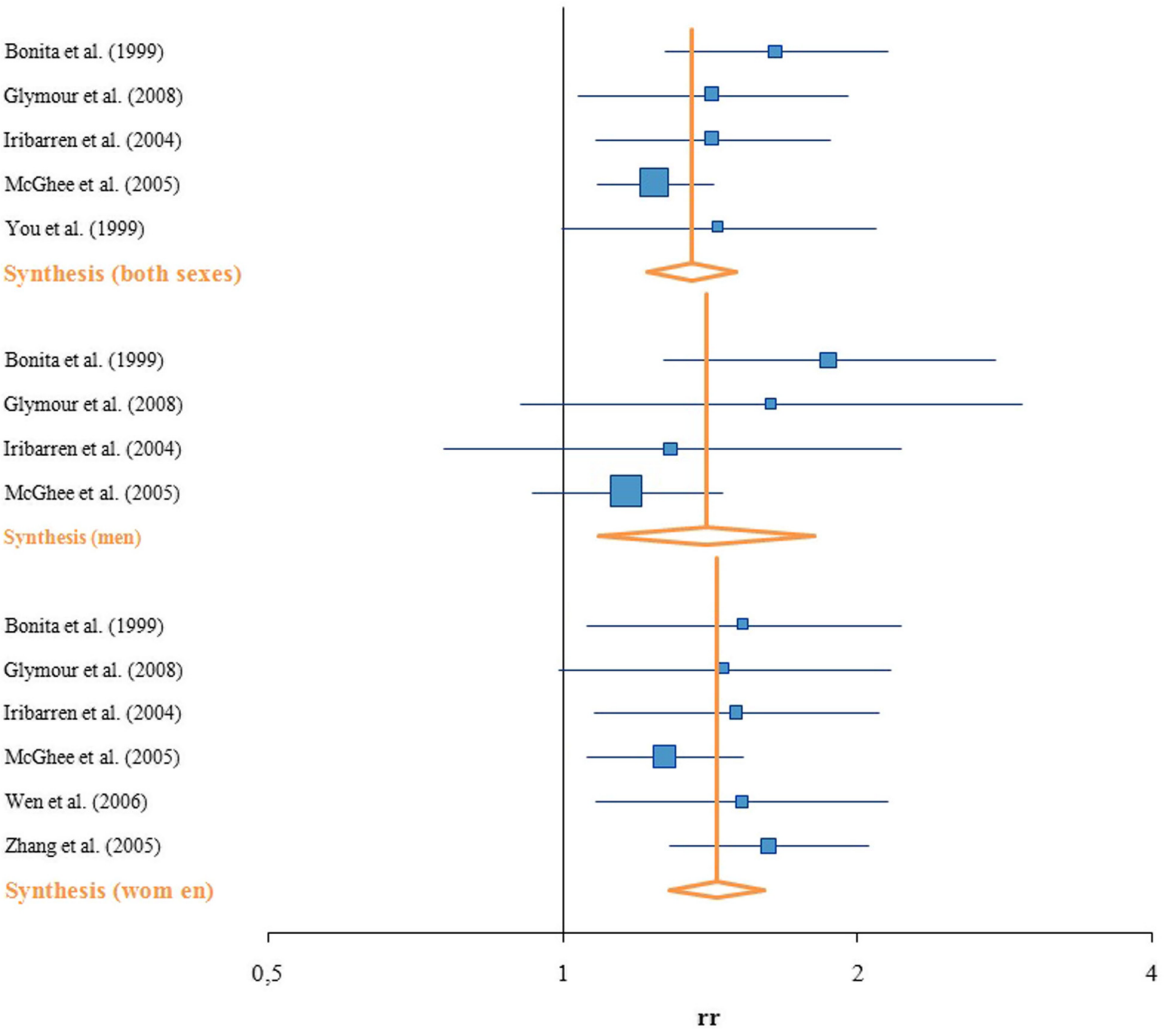
Forest plot–SHS and stroke

The synthesis for the three stroke subgroups differs from the two outcomes for IHD and COPD described above. In this case, the RR for the association between SHS and stroke is 1.35 (95 % CI: 1.22 – 1.50) for both sexes combined. The analysis separated for sex led to a slightly higher RR for men (RR = 1.40, 95 % CI: 1.09–1.81) as well as for women (RR = 1.43, 95 % CI: 1.28–1.61) compared to the synthesis for both sexes (Table 4, Fig. 4). This is due to the fact that the studies included in the meta-analysis in which both sexes are considered in a combined effect size are not exclusively the same as those which show results for men or women separately. One study only gives results for both sexes combined [25] and two studies only give results for women [73, 74].

The Q-statistic indicated no heterogeneity, although the *p*-value for the Q-statistic for men was 0.184 and therefore close to the border indicating heterogeneity. According to the I^2^, the studies for women (I^2^ = 0 %) as well as for both sexes (I^2^ = 2.08 %) are homogeneous. For men, a low to medium heterogeneity was observed (I^2^ = 37.95 %) (Table 4).

## Discussion

In this study, the effect sizes for IHD, COPD and stroke attributable to SHS exposure were estimated. For all three outcomes, the effect sizes were larger for women than for men. In men, statistically significant results were revealed only for the association between SHS exposure and stroke. According to the calculated effect sizes for all three disease entities, the risk factor of SHS exposure seems to be particularly important for COPD. A 66 % excess risk of COPD was calculated for people exposed to SHS for both sexes combined. For stroke (RR = 1.35, 95 % CI: 1.22–1.50) and IHD (RR = 1.27, 95 % CI: 1.10–1.48), the RR was considerably lower.

### IHD

The calculated association between SHS exposure and IHD is consistent with several meta-analyses calculating the overall RR of coronary heart diseases associated with SHS exposure among non-smokers. In a meta-analysis including 18 studies (10 prospective cohort studies and eight case–control studies), the estimated RR was 1.25 (95 % CI: 1.17–1.32) [20]. A meta-analysis by Wells [12] focused on the association between IHD mortality and SHS exposure. According to this study, a RR of 1.23 (95 % CI: 1.12–1.35) was calculated for both sexes combined (men: RR = 1.25, 95 % CI: 1.03–1.51; women: RR = 1.23, 95 % CI: 1.11–1.36) [12]. These estimations are comparable to the calculation of the effect size for both sexes combined. Nevertheless, the study by Wells [12] provided effect sizes which are almost equal for both sexes. In our study the results for the association between SHS exposure and IHD indicated much higher effect sizes for women. Wells [12] also calculated the effect size associating IHD morbidity with SHS exposure. Here, the RR for women was 1.51 (95 % CI: 1.16–1.97), which is comparable to the estimation of the results of our study. Therefore, it seems that the associations for IHD morbidity and mortality differ substantially, and this leads to differences in the effect sizes estimated in this study compared to previous ones.

### COPD

The estimation of the effect size for the association between SHS exposure and COPD cannot be compared to other meta-analyses, because this is the first attempt to calculate a synthesis for the primary studies dealing with this association. Up to now, the number of studies on SHS exposure as a risk factor for adult onset COPD is small compared with the number on the adverse health effects of SHS exposure on childhood respiratory symptoms and diseases [22]. The estimation for both sexes combined led to a RR of 1.66 (95 % CI: 1.38–2.00), which is higher than the estimation for the association between SHS exposure and IHD. This also applies to the gender stratified estimations: in women a RR of 2.17 was calculated with a fairly broad confidence interval (95 % CI: 1.48–3.18). This can be explained by the fact that three of the total of five studies dealt with the association in women. The studies by Wu et al. [75] (RR = 3.12, 95 % CI: 1.56–6.50) and McGhee et al. [24] (RR = 2.59, 95 % CI: 1.30–5.27) in particular contributed to the broad confidence interval. Therefore, the few existing studies on SHS exposure and COPD differ considerably, although the results indicate a positive association. No judgement on the consistency of the results of primary studies on the association between SHS exposure and COPD for men is possible, because only the study by McGhee et al. [24] provided results for the male subgroup (RR = 1.50, 95 % CI: 0.96–2.28).

### Stroke

The estimations for the association between SHS exposure and stroke (RR = 1.35, 95 % CI: 1.22–1.50) are comparable with previous meta-analyses. In our study, the effect sizes showed a significantly increased risk for people exposed to SHS in both sexes, with RRs that are almost equal between men (RR = 1.40, 95 % CI: 1.09–1.81) and women (RR–1.43, 95 % CI: 1.28–1.61). Lee and Forey [76] provided a comprehensive review of epidemiological evidence relating stroke to SHS exposure in lifelong non-smokers. Overall, including 16 studies (seven prospective cohort studies, six case–control studies and three cross-sectional studies) which used current spousal smoking (or nearest equivalent) as the exposure index led to an overall estimate of 1.25 (95 % CI: 1.16–1.36), which is slightly lower than our calculations. The study results also indicated no significant heterogeneity and no differences between men and women [76], which is consistent with our study results. Eight studies in the meta-analysis provided information regarding a possible dose–response relationship between SHS exposure and stroke. According to this, the synthesis for the highest level of exposure led to a RR of 1.56 (95 % CI: 1.34–1.82).

Another meta-analysis [49], included 20 studies (10 cohort studies, six case–control studies and four cross-sectional studies) published between 1984 and 2010. All of these reported results for non-smokers, who were mainly defined as never-smokers, although some studies also included ex-smokers or infrequent current smokers. Eleven studies in the meta-analysis by Oono et al. [49] measured the dose of SHS exposure, which was either defined as the number of smokers, cigarettes per day, hours per week, pack years, or cotinine concentration and score. Our calculations for the effect size of the increased risk of stroke attributable to SHS exposure (RR = 1.35, 95 % CI: 1.22–1.50) are in line with the results of SHS exposure of either 10 cigarettes per day (RR = 1.31, 95 % CI: 1.12–1.54) or 15 cigarettes per day (RR = 1.45, 95 % CI: 1.19–1.78) [49].

### Dose–response relationship

The results of the primary studies that were included in the meta-analysis on the associations between SHS exposure and IHD as well as stroke indicate a distinct dose–response relationship. Even low levels of SHS exposure increase the risk of adverse health effects, indicating that there is no safe level of exposure [42, 49]. The effects of SHS exposure are lower than those of active smoking, but it has been consistently shown that the effects of SHS exposure on the cardiovascular system are much larger than might be expected from a comparison of the doses of toxins delivered to active and passive smokers. Therefore the effects of SHS are estimated to be on average 80-90 % as harmful as those of active smoking [10]. The effects of a dose–response relationship between SHS exposure and adverse health outcomes were not depicted in this study, because it focused on regular exposure to SHS. Although the dose–response function might supply important additional information, Sauerbrei et al. [77] argued that aggregated data are too limited to perform a meta-analysis including a dose–response analysis. Nevertheless, regular SHS exposure, irrespective of the dose is still an important risk factor, because it may lead to both acute and chronic diseases.

### Gender differences

The stratification for sex performed in this study is highly relevant, because the effect sizes as well as the prevalence of diseases and the prevalence of SHS exposure differ between the sexes. Until now, it has been largely men who have been considered in many studies dealing with IHD, because of their higher prevalence of coronary diseases. In most parts of the world women are at least 50 % more likely to be exposed to SHS than men [78]. Until now, only a few studies have investigated possible mechanisms underlying sex differences in adverse health outcomes such as IHD related to SHS exposure. It is assumed that the anti-oestrogenic effect of cigarette smoking–and therefore also the exposure to SHS–may be at least partly related to the increased risk of IHD in young females smokers [79]. Furthermore, a study by Geisler et al. [80] indicated that in smoking women undergoing oestrogen replacement therapy, plasma levels of oestrogen were 40-70 % lower than in non-smoking women. Additionally, a decrease in both oestradiol and testosterone concentrations in smoking men has been reported [81]. Therefore, hormonal factors seem to considerably influence vulnerability due to SHS exposure. This might be one explanation for gender differences in the effects of SHS exposure [82].

### Limitations

There are methodological restrictions in data quality of primary studies, which have to be considered when interpreting the results. Among these, particularly the differences in study designs and misclassification bias due to different definitions and measurements of SHS exposure have to be mentioned. Another limitation of major importance in the context of a systematic literature review is a possible publication bias, although a review of published and unpublished studies on the health effects of SHS exposure showed no evidence of publication bias against statistically non-significant results in the peer-reviewed literature [83].

Another limitation in the identification of primary studies on the association between SHS exposure and the three selected diseases leads back to the decision to perform the systematic literature search only in one literature database, PubMed. Therefore, some studies might have been missed, although an additional manual search in the reference lists of publications was performed, which led to only eight further articles. A broader search strategy with another search algorithm may have led to further articles eligible for the meta-analysis.

The quality assessment led to the exclusion of nine studies. Although the development of criteria for the quality assessment was based on established instruments, different criteria may have led to the exclusion of more or fewer articles, depending on their strictness. The quality checklist was used as a scale, although the criticism has been made that these scales do not provide a transparent estimation of the degree of bias [84]. Furthermore, quality scores neglect information about individual items and no empirical basis for the different weights that are implicitly given to each item exists [85]. Nevertheless, this approach was chosen, to allow for the exclusion of studies with low methodological quality.

Since only cohort studies and case–control studies were selected, a large number of studies had to be excluded either during the screening of titles and abstracts or during the assessment of full-texts. Also, comparatively small studies with low effect sizes or rather broad confidence intervals were included in the meta-analysis. These studies carried a smaller weight in the synthesis of results. To make the results of the primary studies comparable, all the OR provided in case–control studies were re-calculated into RR using quite a conservative approach, which is more likely to underestimate the true association. Therefore, overall, the effect sizes calculated in the meta-analysis represent a conservative estimate.

Besides the identification and data quality of primary studies, the combination of research results from multiple studies performed in the meta-analysis faces several limitations and uncertainties. Although this meta-analysis indicates only low heterogeneity, diversity between studies, for example due to different populations (e.g., countries, age groups), inclusion and exclusion criteria (e.g., more severe patients), study designs (e.g., inadequate follow-up of lost patients), statistical methods used, and various sources of bias, is still an important issue. Formal heterogeneity tests face low statistical power. In this study, the Q-statistic and I^2^-test were used. A shortcoming of the Q-statistic is that it has low power to detect true heterogeneity among studies when the meta-analysis includes only a small number of studies [86]. The Q-statistic is useful to test for the existence of heterogeneity, but not to assess the magnitude of heterogeneity. For that we used the I^2^.

For the calculation of the effect sizes for COPD, it has to be kept in mind that the estimation for men is based on only one study. Particularly for COPD, the synthesis is based on very few studies, which limits its reliability. The combination of studies will often result in small confidence intervals, suggesting a false precision [58]. In this context, it is relevant to point out that random effects models, as used in this study, are not sufficient to explain the heterogeneity between studies, since the random effect merely quantifies an unexplained variation by estimating it [59].

### Conclusion and implications

Up to now, the effects of SHS exposure on population health are still controversial, although several studies and meta-analyses have revealed comparable results on the association between regular SHS exposure and adverse health outcomes. However, further studies with sound methodological approaches due to large prospective epidemiological studies using biomarkers for exposure assessment are still required to determine the risks associated with SHS exposure [16, 76]. Furthermore, there is only a little evidence for the effects of SHS on health-related quality of life, which is a very important parameter for well-being besides objective parameters such as morbidity and mortality.

To address this research need, this study was conducted. It is the first study to have calculated effect sizes for the association between SHS exposure and the disease outcomes IHD, COPD, and stroke, stratified by sex. The effect sizes calculated in the meta-analysis are overall comparable with previous findings in meta-analyses for IHD and stroke. This suggests that the results are reliable. Although no previous meta-analysis for the association between SHS exposure and COPD is available, the results are assumed to be reliable as well, because the methodological approach in this study was the same for all three disease entities. Nevertheless, further research is needed, to provide more adequate primary studies which account for confounding and other biases.

## Additional file

Additional file 1:
**Caption: Heterogeneity funnel plots.** (DOCX 20 kb)

## Footnotes

1 Because some articles dealt with multiple outcomes, the sum of all articles did not add up to 403.

2 The number of articles excluded for each criterion is mentioned in brackets. The sum did not add up to 71, because some articles were excluded for multiple reasons.
