# Supplementary material for: Meta-analysis of the association between second-hand smoke exposure and ischaemic heart diseases, COPD and stroke
Source: BMC Public Health. 2015 Dec 1;15:1202. doi: 10.1186/s12889-015-2489-4 (PMC4667413; doi:10.1186/s12889-015-2489-4)
Supplement: Additional file 1: — Caption: Heterogeneity funnel plots. (DOCX 20 kb) [file 12889_2015_2489_MOESM1_ESM.docx]

**Additional file**

Heterogeneity funnel plot IHD

(black: both sexes; red: women; blue: men)

Figure Heterogeneity funnel plot COPD

(black: both sexes; red: women; blue: men)

Heterogeneity funnel plot stroke

(black: both sexes; red: women; blue: men)
